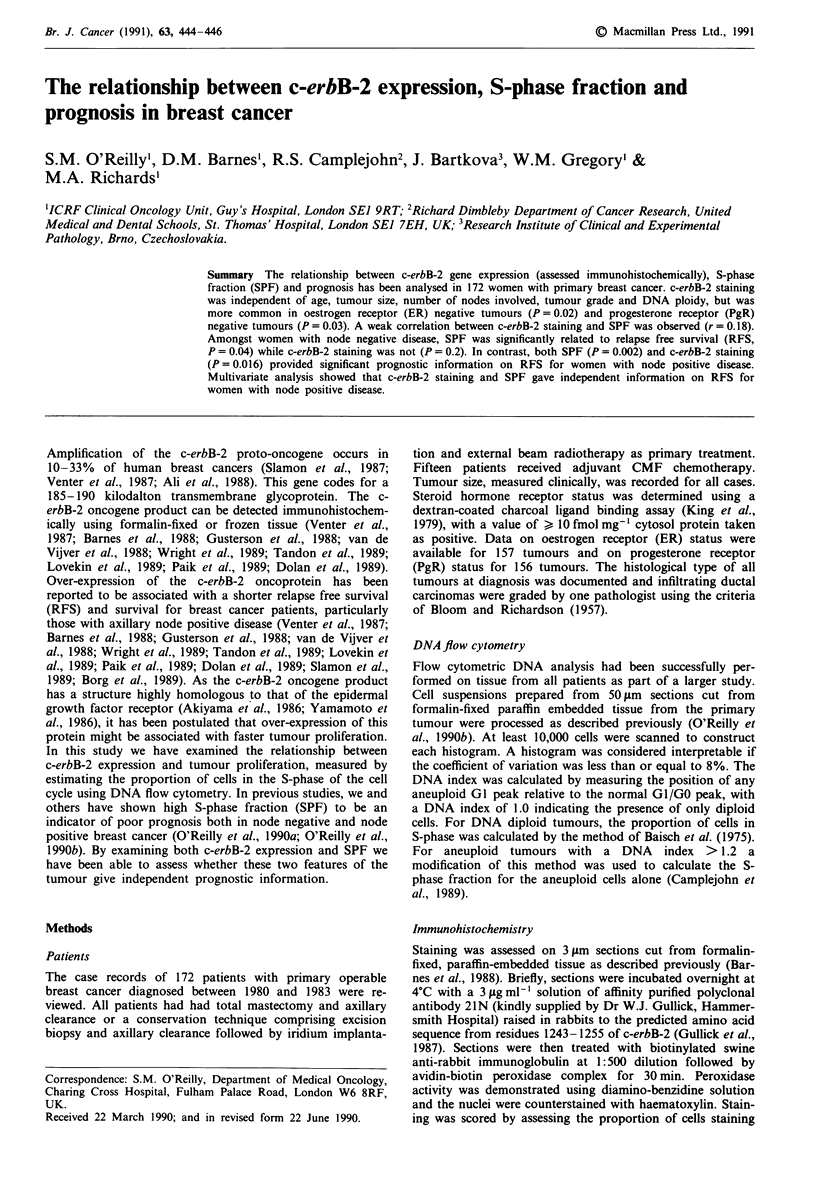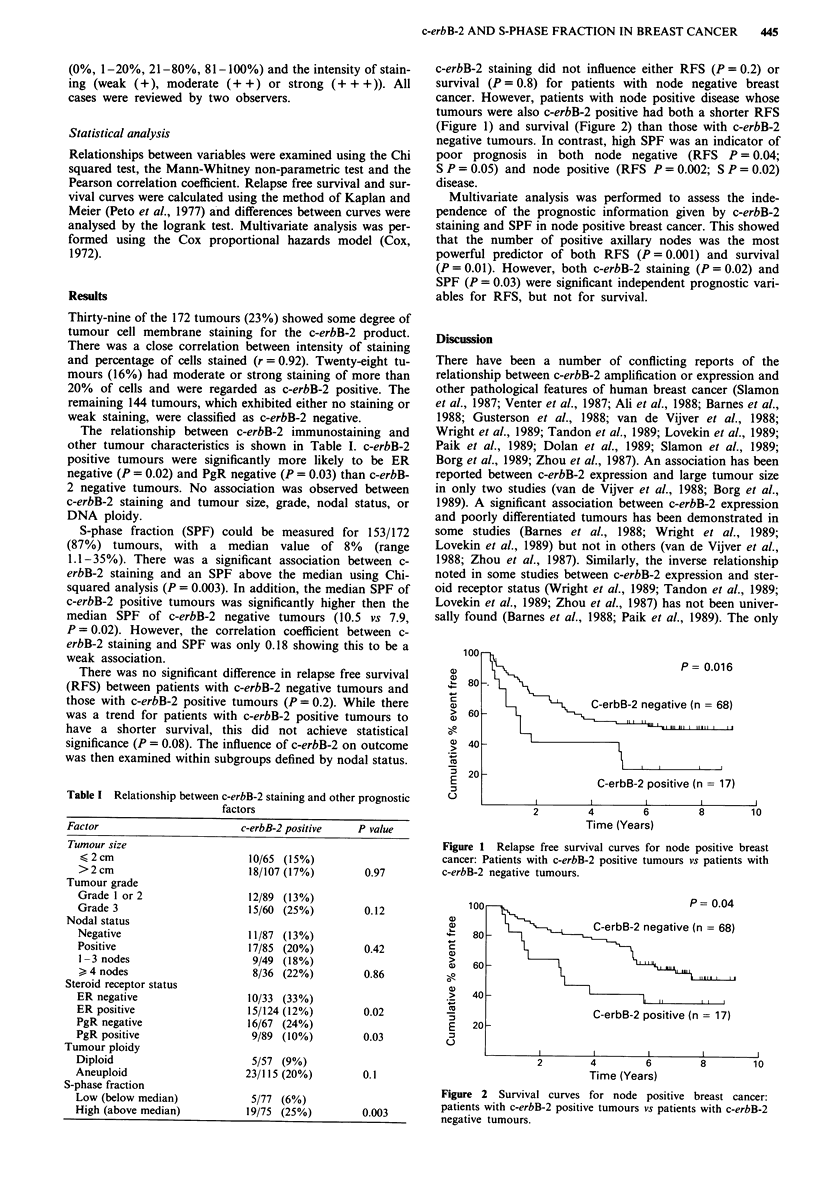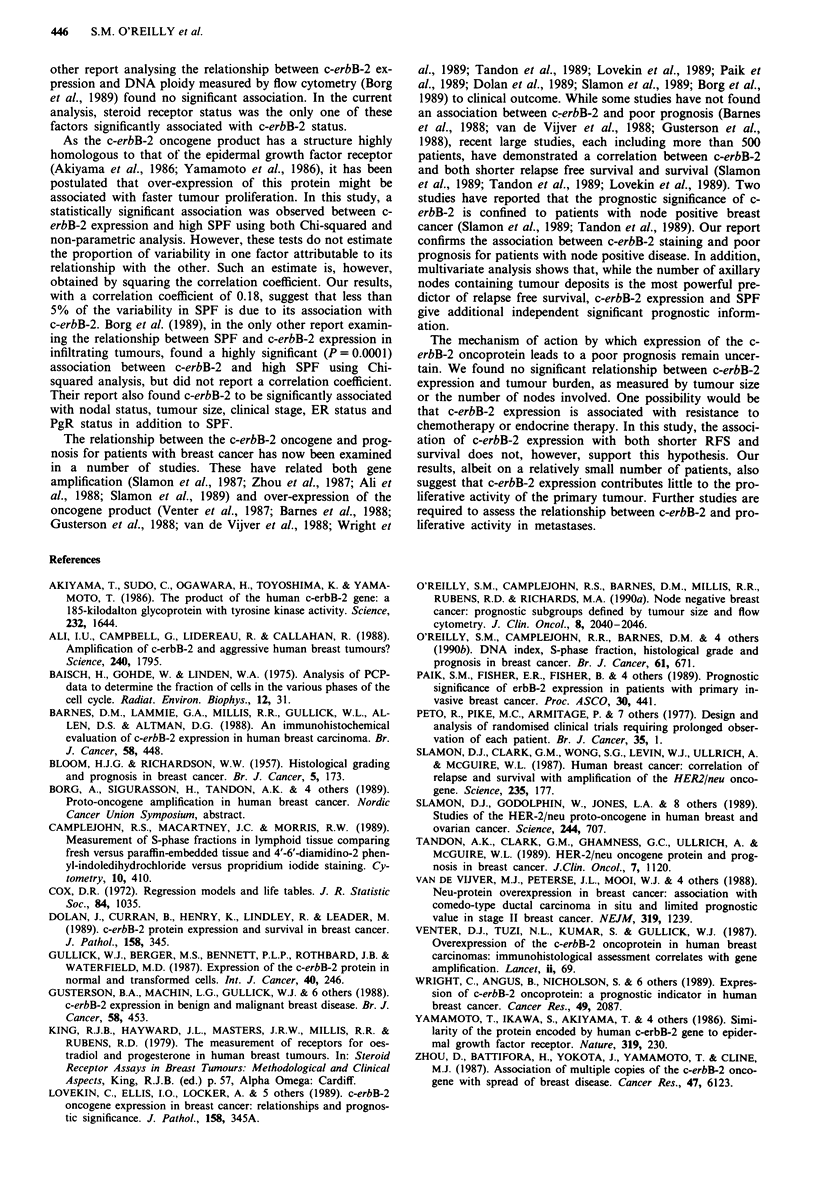# The relationship between c-erbB-2 expression, S-phase fraction and prognosis in breast cancer.

**DOI:** 10.1038/bjc.1991.102

**Published:** 1991-03

**Authors:** S. M. O'Reilly, D. M. Barnes, R. S. Camplejohn, J. Bartkova, W. M. Gregory, M. A. Richards

**Affiliations:** ICRF Clinical Oncology Unit, Guy's Hospital, London, UK.

## Abstract

The relationship between c-erbB-2 gene expression (assessed immunohistochemically), S-phase fraction (SPF) and prognosis has been analysed in 172 women with primary breast cancer. c-erbB-2 staining was independent of age, tumour size, number of nodes involved, tumour grade and DNA ploidy, but was more common in oestrogen receptor (ER) negative tumours (P = 0.02) and progesterone receptor (PgR) negative tumours (P = 0.03). A weak correlation between c-erbB-2 staining and SPF was observed (r = 0.18). Amongst women with node negative disease, SPF was significantly related to relapse free survival (RFS, P = 0.04) while c-erbB-2 staining was not (P = 0.2). In contrast, both SPF (P = 0.002) and c-erbB-2 staining (P = 0.016) provided significant prognostic information on RFS for women with node positive disease. Multivariate analysis showed that c-erbB-2 staining and SPF gave independent information on RFS for women with node positive disease.


Br. J. Cancer (1991), 63, 444-446 © Macmillan Press Ltd., 1991

### The relationship between c-*erb*B-2 expression, S-phase fraction and prognosis in breast cancer

S.M. O'Reilly[1], D.M. Barnes[1], R.S. Camplejohn[2], J. Bartkova[3], W.M. Gregory[1] & M.A. Richards[1]

[1]*ICRF Clinical Oncology Unit, Guy's Hospital, London SE1 9RT;* [2]*Richard Dimbleby Department of Cancer Research, United Medical and Dental Schools, St. Thomas' Hospital, London SE1 7EH, UK;* [3]*Research Institute of Clinical and Experimental Pathology, Brno, Czechoslovakia.*

**Summary** The relationship between c-*erb*B-2 gene expression (assessed immunohistochemically), S-phase fraction (SPF) and prognosis has been analysed in 172 women with primary breast cancer. c-*erb*B-2 staining was independent of age, tumour size, number of nodes involved, tumour grade and DNA ploidy, but was more common in oestrogen receptor (ER) negative tumours ($P = 0.02$) and progesterone receptor (PgR) negative tumours ($P = 0.03$). A weak correlation between c-*erb*B-2 staining and SPF was observed ($r = 0.18$). Amongst women with node negative disease, SPF was significantly related to relapse free survival (RFS, $P = 0.04$) while c-*erb*B-2 staining was not ($P = 0.2$). In contrast, both SPF ($P = 0.002$) and c-*erb*B-2 staining ($P = 0.016$) provided significant prognostic information on RFS for women with node positive disease. Multivariate analysis showed that c-*erb*B-2 staining and SPF gave independent information on RFS for women with node positive disease.

Amplification of the c-*erb*B-2 proto-oncogene occurs in 10-33% of human breast cancers (Slamon *et al.*, 1987; Venter *et al.*, 1987; Ali *et al.*, 1988). This gene codes for a 185-190 kilodalton transmembrane glycoprotein. The c-*erb*B-2 oncogene product can be detected immunohistochemically using formalin-fixed or frozen tissue (Venter *et al.*, 1987; Barnes *et al.*, 1988; Gusterson *et al.*, 1988; van de Vijver *et al.*, 1988; Wright *et al.*, 1989; Tandon *et al.*, 1989; Lovekin *et al.*, 1989; Paik *et al.*, 1989; Dolan *et al.*, 1989). Over-expression of the c-*erb*B-2 oncoprotein has been reported to be associated with a shorter relapse free survival (RFS) and survival for breast cancer patients, particularly those with axillary node positive disease (Venter *et al.*, 1987; Barnes *et al.*, 1988; Gusterson *et al.*, 1988; van de Vijver *et al.*, 1988; Wright *et al.*, 1989; Tandon *et al.*, 1989; Lovekin *et al.*, 1989; Paik *et al.*, 1989; Dolan *et al.*, 1989; Slamon *et al.*, 1989; Borg *et al.*, 1989). As the c-*erb*B-2 oncogene product has a structure highly homologous to that of the epidermal growth factor receptor (Akiyama *et al.*, 1986; Yamamoto *et al.*, 1986), it has been postulated that over-expression of this protein might be associated with faster tumour proliferation. In this study we have examined the relationship between c-*erb*B-2 expression and tumour proliferation, measured by estimating the proportion of cells in the S-phase of the cell cycle using DNA flow cytometry. In previous studies, we and others have shown high S-phase fraction (SPF) to be an indicator of poor prognosis both in node negative and node positive breast cancer (O'Reilly *et al.*, 1990*a*; O'Reilly *et al.*, 1990*b*). By examining both c-*erb*B-2 expression and SPF we have been able to assess whether these two features of the tumour give independent prognostic information.

### Methods

### *Patients*

The case records of 172 patients with primary operable breast cancer diagnosed between 1980 and 1983 were reviewed. All patients had had total mastectomy and axillary clearance or a conservation technique comprising excision biopsy and axillary clearance followed by iridium implantation and external beam radiotherapy as primary treatment. Fifteen patients received adjuvant CMF chemotherapy. Tumour size, measured clinically, was recorded for all cases. Steroid hormone receptor status was determined using a dextran-coated charcoal ligand binding assay (King *et al.*, 1979), with a value of $\geqslant 10\,\text{fmol mg}^{-1}$ cytosol protein taken as positive. Data on oestrogen receptor (ER) status were available for 157 tumours and on progesterone receptor (PgR) status for 156 tumours. The histological type of all tumours at diagnosis was documented and infiltrating ductal carcinomas were graded by one pathologist using the criteria of Bloom and Richardson (1957).

### *DNA flow cytometry*

Flow cytometric DNA analysis had been successfully performed on tissue from all patients as part of a larger study. Cell suspensions prepared from 50 µm sections cut from formalin-fixed paraffin embedded tissue from the primary tumour were processed as described previously (O'Reilly *et al.*, 1990*b*). At least 10,000 cells were scanned to construct each histogram. A histogram was considered interpretable if the coefficient of variation was less than or equal to 8%. The DNA index was calculated by measuring the position of any aneuploid G1 peak relative to the normal G1/G0 peak, with a DNA index of 1.0 indicating the presence of only diploid cells. For DNA diploid tumours, the proportion of cells in S-phase was calculated by the method of Baisch *et al.* (1975). For aneuploid tumours with a DNA index $>1.2$ a modification of this method was used to calculate the S-phase fraction for the aneuploid cells alone (Camplejohn *et al.*, 1989).

### *Immunohistochemistry*

Staining was assessed on 3 µm sections cut from formalin-fixed, paraffin-embedded tissue as described previously (Barnes *et al.*, 1988). Briefly, sections were incubated overnight at 4°C with a $3\,\mu\text{g ml}^{-1}$ solution of affinity purified polyclonal antibody 21N (kindly supplied by Dr W.J. Gullick, Hammersmith Hospital) raised in rabbits to the predicted amino acid sequence from residues 1243-1255 of c-*erb*B-2 (Gullick *et al.*, 1987). Sections were then treated with biotinylated swine anti-rabbit immunoglobulin at 1:500 dilution followed by avidin-biotin peroxidase complex for 30 min. Peroxidase activity was demonstrated using diamino-benzidine solution and the nuclei were counterstained with haematoxylin. Staining was scored by assessing the proportion of cells staining

Correspondence: S.M. O'Reilly, Department of Medical Oncology, Charing Cross Hospital, Fulham Palace Road, London W6 8RF, UK.
Received 22 March 1990; and in revised form 22 June 1990.

(0%, 1–20%, 21–80%, 81–100%) and the intensity of staining (weak (+), moderate (++) or strong (+++)). All cases were reviewed by two observers.

*Statistical analysis*

Relationships between variables were examined using the Chi squared test, the Mann-Whitney non-parametric test and the Pearson correlation coefficient. Relapse free survival and survival curves were calculated using the method of Kaplan and Meier (Peto *et al.*, 1977) and differences between curves were analysed by the logrank test. Multivariate analysis was performed using the Cox proportional hazards model (Cox, 1972).

### Results

Thirty-nine of the 172 tumours (23%) showed some degree of tumour cell membrane staining for the c-*erb*B-2 product. There was a close correlation between intensity of staining and percentage of cells stained ($r = 0.92$). Twenty-eight tumours (16%) had moderate or strong staining of more than 20% of cells and were regarded as c-*erb*B-2 positive. The remaining 144 tumours, which exhibited either no staining or weak staining, were classified as c-*erb*B-2 negative.

The relationship between c-*erb*B-2 immunostaining and other tumour characteristics is shown in Table I. c-*erb*B-2 positive tumours were significantly more likely to be ER negative ($P = 0.02$) and PgR negative ($P = 0.03$) than c-*erb*B-2 negative tumours. No association was observed between c-*erb*B-2 staining and tumour size, grade, nodal status, or DNA ploidy.

S-phase fraction (SPF) could be measured for 153/172 (87%) tumours, with a median value of 8% (range 1.1–35%). There was a significant association between c-*erb*B-2 staining and an SPF above the median using Chi-squared analysis ($P = 0.003$). In addition, the median SPF of c-*erb*B-2 positive tumours was significantly higher then the median SPF of c-*erb*B-2 negative tumours (10.5 *vs* 7.9, $P = 0.02$). However, the correlation coefficient between c-*erb*B-2 staining and SPF was only 0.18 showing this to be a weak association.

There was no significant difference in relapse free survival (RFS) between patients with c-*erb*B-2 negative tumours and those with c-*erb*B-2 positive tumours ($P = 0.2$). While there was a trend for patients with c-*erb*B-2 positive tumours to have a shorter survival, this did not achieve statistical significance ($P = 0.08$). The influence of c-*erb*B-2 on outcome was then examined within subgroups defined by nodal status.

**Table I** Relationship between c-*erb*B-2 staining and other prognostic factors

| *Factor* | *c-erbB-2 positive* | *P value* |
|---|---|---|
| *Tumour size* | | |
| ≤2 cm | 10/65 (15%) | |
| >2 cm | 18/107 (17%) | 0.97 |
| Tumour grade | | |
| Grade 1 or 2 | 12/89 (13%) | |
| Grade 3 | 15/60 (25%) | 0.12 |
| Nodal status | | |
| Negative | 11/87 (13%) | |
| Positive | 17/85 (20%) | 0.42 |
| 1–3 nodes | 9/49 (18%) | |
| ≥4 nodes | 8/36 (22%) | 0.86 |
| Steroid receptor status | | |
| ER negative | 10/33 (33%) | |
| ER positive | 15/124 (12%) | 0.02 |
| PgR negative | 16/67 (24%) | |
| PgR positive | 9/89 (10%) | 0.03 |
| Tumour ploidy | | |
| Diploid | 5/57 (9%) | |
| Aneuploid | 23/115 (20%) | 0.1 |
| S-phase fraction | | |
| Low (below median) | 5/77 (6%) | |
| High (above median) | 19/75 (25%) | 0.003 |

c-*erb*B-2 staining did not influence either RFS ($P = 0.2$) or survival ($P = 0.8$) for patients with node negative breast cancer. However, patients with node positive disease whose tumours were also c-*erb*B-2 positive had both a shorter RFS (Figure 1) and survival (Figure 2) than those with c-*erb*B-2 negative tumours. In contrast, high SPF was an indicator of poor prognosis in both node negative (RFS $P = 0.04$; S $P = 0.05$) and node positive (RFS $P = 0.002$; S $P = 0.02$) disease.

Multivariate analysis was performed to assess the independence of the prognostic information given by c-*erb*B-2 staining and SPF in node positive breast cancer. This showed that the number of positive axillary nodes was the most powerful predictor of both RFS ($P = 0.001$) and survival ($P = 0.01$). However, both c-*erb*B-2 staining ($P = 0.02$) and SPF ($P = 0.03$) were significant independent prognostic variables for RFS, but not for survival.

### Discussion

There have been a number of conflicting reports of the relationship between c-*erb*B-2 amplification or expression and other pathological features of human breast cancer (Slamon *et al.*, 1987; Venter *et al.*, 1987; Ali *et al.*, 1988; Barnes *et al.*, 1988; Gusterson *et al.*, 1988; van de Vijver *et al.*, 1988; Wright *et al.*, 1989; Tandon *et al.*, 1989; Lovekin *et al.*, 1989; Paik *et al.*, 1989; Dolan *et al.*, 1989; Slamon *et al.*, 1989; Borg *et al.*, 1989; Zhou *et al.*, 1987). An association has been reported between c-*erb*B-2 expression and large tumour size in only two studies (van de Vijver *et al.*, 1988; Borg *et al.*, 1989). A significant association between c-*erb*B-2 expression and poorly differentiated tumours has been demonstrated in some studies (Barnes *et al.*, 1988; Wright *et al.*, 1989; Lovekin *et al.*, 1989) but not in others (van de Vijver *et al.*, 1988; Zhou *et al.*, 1987). Similarly, the inverse relationship noted in some studies between c-*erb*B-2 expression and steroid receptor status (Wright *et al.*, 1989; Tandon *et al.*, 1989; Lovekin *et al.*, 1989; Zhou *et al.*, 1987) has not been universally found (Barnes *et al.*, 1988; Paik *et al.*, 1989). The only

**Figure 1** Relapse free survival curves for node positive breast cancer: Patients with c-*erb*B-2 positive tumours *vs* patients with c-*erb*B-2 negative tumours.

**Figure 2** Survival curves for node positive breast cancer: patients with c-*erb*B-2 positive tumours *vs* patients with c-*erb*B-2 negative tumours.

other report analysing the relationship between c-*erb*B-2 expression and DNA ploidy measured by flow cytometry (Borg *et al.*, 1989) found no significant association. In the current analysis, steroid receptor status was the only one of these factors significantly associated with c-*erb*B-2 status.

As the c-*erb*B-2 oncogene product has a structure highly homologous to that of the epidermal growth factor receptor (Akiyama *et al.*, 1986; Yamamoto *et al.*, 1986), it has been postulated that over-expression of this protein might be associated with faster tumour proliferation. In this study, a statistically significant association was observed between c-*erb*B-2 expression and high SPF using both Chi-squared and non-parametric analysis. However, these tests do not estimate the proportion of variability in one factor attributable to its relationship with the other. Such an estimate is, however, obtained by squaring the correlation coefficient. Our results, with a correlation coefficient of 0.18, suggest that less than 5% of the variability in SPF is due to its association with c-*erb*B-2. Borg *et al.* (1989), in the only other report examining the relationship between SPF and c-*erb*B-2 expression in infiltrating tumours, found a highly significant ($P = 0.0001$) association between c-*erb*B-2 and high SPF using Chi-squared analysis, but did not report a correlation coefficient. Their report also found c-*erb*B-2 to be significantly associated with nodal status, tumour size, clinical stage, ER status and PgR status in addition to SPF.

The relationship between the c-*erb*B-2 oncogene and prognosis for patients with breast cancer has now been examined in a number of studies. These have related both gene amplification (Slamon *et al.*, 1987; Zhou *et al.*, 1987; Ali *et al.*, 1988; Slamon *et al.*, 1989) and over-expression of the oncogene product (Venter *et al.*, 1987; Barnes *et al.*, 1988; Gusterson *et al.*, 1988; van de Vijver *et al.*, 1988; Wright *et al.*, 1989; Tandon *et al.*, 1989; Lovekin *et al.*, 1989; Paik *et al.*, 1989; Dolan *et al.*, 1989; Slamon *et al.*, 1989; Borg *et al.*, 1989) to clinical outcome. While some studies have not found an association between c-*erb*B-2 and poor prognosis (Barnes *et al.*, 1988; van de Vijver *et al.*, 1988; Gusterson *et al.*, 1988), recent large studies, each including more than 500 patients, have demonstrated a correlation between c-*erb*B-2 and both shorter relapse free survival and survival (Slamon *et al.*, 1989; Tandon *et al.*, 1989; Lovekin *et al.*, 1989). Two studies have reported that the prognostic significance of c-*erb*B-2 is confined to patients with node positive breast cancer (Slamon *et al.*, 1989; Tandon *et al.*, 1989). Our report confirms the association between c-*erb*B-2 staining and poor prognosis for patients with node positive disease. In addition, multivariate analysis shows that, while the number of axillary nodes containing tumour deposits is the most powerful predictor of relapse free survival, c-*erb*B-2 expression and SPF give additional independent significant prognostic information.

The mechanism of action by which expression of the c-*erb*B-2 oncoprotein leads to a poor prognosis remain uncertain. We found no significant relationship between c-*erb*B-2 expression and tumour burden, as measured by tumour size or the number of nodes involved. One possibility would be that c-*erb*B-2 expression is associated with resistance to chemotherapy or endocrine therapy. In this study, the association of c-*erb*B-2 expression with both shorter RFS and survival does not, however, support this hypothesis. Our results, albeit on a relatively small number of patients, also suggest that c-*erb*B-2 expression contributes little to the proliferative activity of the primary tumour. Further studies are required to assess the relationship between c-*erb*B-2 and proliferative activity in metastases.

### References

AKIYAMA, T., SUDO, C., OGAWARA, H., TOYOSHIMA, K. & YAMAMOTO, T. (1986). The product of the human c-erbB-2 gene: a 185-kilodalton glycoprotein with tyrosine kinase activity. *Science*, **232**, 1644.

ALI, I.U., CAMPBELL, G., LIDEREAU, R. & CALLAHAN, R. (1988). Amplification of c-erbB-2 and aggressive human breast tumours? *Science*, **240**, 1795.

BAISCH, H., GOHDE, W. & LINDEN, W.A. (1975). Analysis of PCP-data to determine the fraction of cells in the various phases of the cell cycle. *Radiat. Environ. Biophys.*, **12**, 31.

BARNES, D.M., LAMMIE, G.A., MILLIS, R.R., GULLICK, W.L., ALLEN, D.S. & ALTMAN, D.G. (1988). An immunohistochemical evaluation of c-*erb*B-2 expression in human breast carcinoma. *Br. J. Cancer*, **58**, 448.

BLOOM, H.J.G. & RICHARDSON, W.W. (1957). Histological grading and prognosis in breast cancer. *Br. J. Cancer*, **5**, 173.

BORG, A., SIGURASSON, H., TANDON, A.K. & 4 others (1989). Proto-oncogene amplification in human breast cancer. *Nordic Cancer Union Symposium*, abstract.

CAMPLEJOHN, R.S., MACARTNEY, J.C. & MORRIS, R.W. (1989). Measurement of S-phase fractions in lymphoid tissue comparing fresh versus paraffin-embedded tissue and 4'-6'-diamidino-2 phenyl-indoledihydrochloride versus propridium iodide staining. *Cytometry*, **10**, 410.

COX, D.R. (1972). Regression models and life tables. *J. R. Statistic Soc.*, **84**, 1035.

DOLAN, J., CURRAN, B., HENRY, K., LINDLEY, R. & LEADER, M. (1989). c-*erb*B-2 protein expression and survival in breast cancer. *J. Pathol.*, **158**, 345.

GULLICK, W.J., BERGER, M.S., BENNETT, P.L.P., ROTHBARD, J.B. & WATERFIELD, M.D. (1987). Expression of the c-*erb*B-2 protein in normal and transformed cells. *Int. J. Cancer*, **40**, 246.

GUSTERSON, B.A., MACHIN, L.G., GULLICK, W.J. & 6 others (1988). c-*erb*B-2 expression in benign and malignant breast disease. *Br. J. Cancer*, **58**, 453.

KING, R.J.B., HAYWARD, J.L., MASTERS, J.R.W., MILLIS, R.R. & RUBENS, R.D. (1979). The measurement of receptors for oestradiol and progesterone in human breast tumours. In: *Steroid Receptor Assays in Breast Tumours: Methodological and Clinical Aspects*, King, R.J.B. (ed.) p. 57, Alpha Omega: Cardiff.

LOVEKIN, C., ELLIS, I.O., LOCKER, A. & 5 others (1989). c-*erb*B-2 oncogene expression in breast cancer: relationships and prognostic significance. *J. Pathol.*, **158**, 345A.

O'REILLY, S.M., CAMPLEJOHN, R.S., BARNES, D.M., MILLIS, R.R., RUBENS, R.D. & RICHARDS, M.A. (1990*a*). Node negative breast cancer: prognostic subgroups defined by tumour size and flow cytometry. *J. Clin. Oncol.*, **8**, 2040–2046.

O'REILLY, S.M., CAMPLEJOHN, R.R., BARNES, D.M. & 4 others (1990*b*). DNA index, S-phase fraction, histological grade and prognosis in breast cancer. *Br. J. Cancer*, **61**, 671.

PAIK, S.M., FISHER, E.R., FISHER, B. & 4 others (1989). Prognostic significance of erbB-2 expression in patients with primary invasive breast cancer. *Proc. ASCO*, **30**, 441.

PETO, R., PIKE, M.C., ARMITAGE, P. & 7 others (1977). Design and analysis of randomised clinical trials requiring prolonged observation of each patient. *Br. J. Cancer*, **35**, 1.

SLAMON, D.J., CLARK, G.M., WONG, S.G., LEVIN, W.J., ULLRICH, A. & MCGUIRE, W.L. (1987). Human breast cancer: correlation of relapse and survival with amplification of the *HER2/neu* oncogene. *Science*, **235**, 177.

SLAMON, D.J., GODOLPHIN, W., JONES, L.A. & 8 others (1989). Studies of the HER-2/neu proto-oncogene in human breast and ovarian cancer. *Science*, **244**, 707.

TANDON, A.K., CLARK, G.M., GHAMNESS, G.C., ULLRICH, A. & MCGUIRE, W.L. (1989). HER-2/neu oncogene protein and prognosis in breast cancer. *J.Clin. Oncol.*, **7**, 1120.

VAN DE VIJVER, M.J., PETERSE, J.L., MOOI, W.J. & 4 others (1988). Neu-protein overexpression in breast cancer: association with comedo-type ductal carcinoma in situ and limited prognostic value in stage II breast cancer. *NEJM*, **319**, 1239.

VENTER, D.J., TUZI, N.L., KUMAR, S. & GULLICK, W.J. (1987). Overexpression of the c-*erb*B-2 oncoprotein in human breast carcinomas: immunohistological assessment correlates with gene amplification. *Lancet*, **ii**, 69.

WRIGHT, C., ANGUS, B., NICHOLSON, S. & 6 others (1989). Expression of c-*erb*B-2 oncoprotein: a prognostic indicator in human breast cancer. *Cancer Res.*, **49**, 2087.

YAMAMOTO, T., IKAWA, S., AKIYAMA, T. & 4 others (1986). Similarity of the protein encoded by human c-erbB-2 gene to epidermal growth factor receptor. *Nature*, **319**, 230.

ZHOU, D., BATTIFORA, H., YOKOTA, J., YAMAMOTO, T. & CLINE, M.J. (1987). Association of multiple copies of the c-*erb*B-2 oncogene with spread of breast disease. *Cancer Res.*, **47**, 6123.